# The population approach to falls injury prevention in older people: findings of a two community trial

**DOI:** 10.1186/1471-2458-10-79

**Published:** 2010-02-19

**Authors:** Rod J McClure, Karen Hughes, Cizao Ren, Kirsten McKenzie, Uta Dietrich, Paul Vardon, Elizabeth Davis, Beth Newman

**Affiliations:** 1 Accident Research Centre, Monash University, Melbourne, Australia; 2 Queensland University of Technology, Brisbane, Australia; 3 North Coast Area Health Service, Lismore, Australia; 4 Queensland Health, Brisbane, Australia; 5 Injury Prevention and Control (Australia) Ltd, Brisbane, Australia

## Abstract

**Background:**

There is a sound rationale for the population-based approach to falls injury prevention but there is currently insufficient evidence to advise governments and communities on how they can use population-based strategies to achieve desired reductions in the burden of falls-related injury. The aim of the study was to quantify the effectiveness of a streamlined (and thus potentially sustainable and cost-effective), population-based, multi-factorial falls injury prevention program for people over 60 years of age.

**Methods:**

Population-based falls-prevention interventions were conducted at two geographically-defined and separate Australian sites: Wide Bay, Queensland, and Northern Rivers, NSW. Changes in the prevalence of key risk factors and changes in rates of injury outcomes within each community were compared before and after program implementation and changes in rates of injury outcomes in each community were also compared with the rates in their respective States.

**Results:**

The interventions in neither community substantially decreased the rate of falls-related injury among people aged 60 years or older, although there was some evidence of reductions in occurrence of multiple falls reported by women. In addition, there was some indication of improvements in fall-related risk factors, but the magnitudes were generally modest.

**Conclusions:**

The evidence suggests that low intensity population-based falls prevention programs may not be as effective as those that are intensively implemented.

## Background

Approximately 28-35% of community-dwelling adults aged 65 years or older will fall each year [1]. Prospective studies have reported that 30% to 60% of older adults residing in the community fall each year, with approximately half of them experiencing multiple falls [2]. Five to 10% of community-dwelling older fallers will sustain a serious injury [3]. Of those admitted to hospital, approximately 50% are subsequently discharged to a nursing home facility [4]. Permanent disability is reported in 32% to 80% of hospitalised cases [5]. Among community-dwelling individuals with fall-related hip fractures, between 25% and 75% do not recover their pre-fracture level of function in ambulation and activities of daily living [6], and remain at high risk for subsequent falls and second fracture [7]. Approximately 25% of fallers restrict their usual activities because of injury or fear of falling again [8].

Because the risk factors for falls among older persons are largely modifiable, preventive interventions have great potential to reduce the rate of falls and subsequent health costs. Interventions will not be effective if targeting high-risk subgroups alone, because most fall-related risk factors are relatively common and the incidence of falls and related injury is also substantial among otherwise healthy older people [9-11]. As the likelihood of falling increases with the number of risk factors, a preventive intervention targeting multiple risk factors has been recommended for use with large populations [12-14]. Cost-effective, population-based programs are needed that can target the broader population and become embedded within the social and physical structures of community function [2,15].

Although community- or population-based, multi-strategy falls-prevention programs have been strongly advocated, there is limited high-quality research evaluating their efficacy [2]. A Cochrane review [2] identified only five studies that met review criteria, i.e., studies reporting changes in medically treated fall-related injuries among older people following the implementation of a controlled, population-based intervention (a coordinated program using multi-strategy initiatives). The authors concluded that population-based, multi-strategy interventions were effective, with programs reporting significant decreases or downward trends in fall-related injuries ranging from six to 33%. They recommended that more quality studies were required to strengthen this result.

The "Stay on Your Feet" (SOYF) program (1992-1995) is the only Australian study to date to report a rigorous evaluation [12]. SOYF was a multi-strategic, population-based, falls-prevention program targeting residents aged 60 years or older and living in North Coast, New South Wales (NSW). Following intervention, there was a 22% lower incidence of self-reported falls in the intervention community compared to a control community. Further, there was a statistically significant 20% lower fall-related hospitalisation rate in the intervention community, and this appears to have been sustained beyond the life of the project [12]. The original SOYF program was extremely resource intensive, utilising a wide range of strategies to address the risk factors for falls among older people. These strategies included awareness raising, community education, local government policy development, home modification, and health professional partnerships. Targeted risk factors included: unsafe footwear, poor vision, lack of exercise, poor balance, inadequate management of medications and chronic health-related conditions, unsafe home environments and unsafe external environments.

The aim of our follow-up research was to evaluate whether a less ambitious, more streamlined (and potentially more sustainable and cost-effective), population-based, multi-factorial intervention could deliver comparable outcomes.

## Methods

### The communities and participants

Community-based falls-prevention interventions were conducted at two geographically-defined and separate Australian sites: Wide Bay (Wide Bay), Queensland (QLD), and Northern Rivers (Northern Rivers), NSW. Community-dwelling residents aged 60 years or older were targeted by the interventions. At the commencement of the study, there were 43,821 (21.6% of total population) and 58,722 (22.2%) residents aged 60+ years living in Wide Bay and Northern Rivers, respectively (data from Australian Bureau of Statistics). Northern Rivers was the site of a prior, multi-strategic falls prevention program, *Stay On Your Feet (SOYF)*, operating during 1992 to 1995 ^13 ^and Wide Bay was essentially a 'greenfield' site.

### The intervention

Increasing physical activity and raising awareness that falls are preventable were the priority strategies selected for the streamlined intervention after an examination of three major sources of information: (1) a sustainability analysis of the 1992-1995 SOYF program; (2) outcomes from a baseline survey of community residents aged 60 years or older; and (3) extensive literature review [13-19]. Both programs used the motto **'***Stay Active, Stay Independent, Stay on Your Feet*', with the project called *'Stay on Your Feet' *(or SOYF) in Wide Bay, but *'Stay Active, Stay Independent' *(or SASI) in Northern Rivers. The goal of the programs was to reduce fall-related injury by introducing and facilitating the incorporation of falls-prevention strategies into existing community structure and services. The implementation of these strategies varied across sites and generally took place between mid-2002 and early 2006.

#### Wide Bay

The approach was similar to that implemented through the original 'Stay on Your Feet' project in that it sought to promote a falls-prevention message within a strong, active ageing context, and to intervene on a wide range of fall-related risk factors while maintaining a emphasis on physical activity. Other strategies focused on safe footwear, home modification, medication review, and public safety. It enlisted a community-based participatory research and practice model, working with local planning groups and a wide range of partners, including community organisations, health professionals, and local and state government departments, to develop and implement local prevention plans. Volunteer, older community members, known as Ambassadors, were trained to deliver these messages and to encourage attitudinal and behavioural changes. Details can be obtained by visiting the program website at http://www.health.qld.gov.au/stayonyourfeet.

#### Northern Rivers

In contrast, Northern Rivers applied a "top-down" approach, working with a well-developed Area Health Service infrastructure including health promotion officers delivering and supporting the interventions, which were focused solely on the priority strategies of facilitating uptake of physical activity to encourage healthy ageing. Activities included 'Come n Try' physical activity expos, Fit to Function volunteer leader training, a multi-media campaign, a general practitioner referral trial, and professional leader training. Details can be obtained by visiting their respective websites at http://www.ncahs.nsw.gov.au/falls.

### Evaluation and analysis

#### Mortality and hospital separations

Multiple causes of death (1997-2004) data were obtained from the Australian Bureau of Statistics and hospital separations (1996-2005) data were obtained from the Australian Institute of Health and Welfare. Data were aggregated by gender (male, female), geographic regions, and 5-year age-groups from 60-64 to 80+ years for morbidity and 85+ years for mortality. Fall-related morbidity and mortality data met either of the following criteria: a) the presence of ICD-10-external cause codes in the range W00-W19 (Accidental Falls) anywhere in either the diagnosis or cause of death strings; or b) the presence of ICD-10 X59 code (Exposure to Unspecified Factor) in combination with an ICD-10-AM fracture injury code anywhere in either the diagnosis or cause of death strings (ICD-10-AM codes S02, S12, S22, S32, S42, S52, S62, S72, S82, S92, T02, T08, T10, T12, T14.2) [20].

For 1996 and 1997, ICD-9-CM was used in Australia, which was backward-mapped from ICD-10-AM codes using tables available from http://www3.fhs.usyd.edu.au/ncchwww/site/4.5.htm. For years with partial information, data were imputed because rates were reasonably stable over short timeframes and this minimised loss of follow-up time. Rates were standardised using the average population of Australia between 1997 and 2004 for mortality and between 1996 and 2005 for incidence[21]. Changes across time in deaths and hospitalisations were analysed comparing Wide Bay and Northern Rivers trends with respect to state-wide trends [22].

#### Baseline and post-intervention telephone survey

Computer-assisted telephone interviews (CATI) were conducted at baseline and post-intervention between February and April in 2002 and 2006, respectively. Members of the community, aged 60 years or older, were targeted, and the survey design ensured equal representation by gender (50% males, 50% females) and intervention region (50% Northern Rivers, 50% Wide Bay). Sampling procedures varied slightly between surveys but both involved random selection of telephone numbers from electronic White Pages, with modifications to ensure inclusion of silent numbers. A telephone number was attempted six times or until contact was made. People were excluded from each of the surveys if they had a hearing disability, did not speak English, or displayed an obvious cognitive disability.

The structured interview instrument was based on items from a mail-out survey utilised in the evaluation of the original SOYF program and several other falls-prevention measures [23-25]. Physical activity questions were adapted from the U.S. Behavioral Risk Factor Surveillance System (BRFSS). ^27 ^Items addressing fall outcomes measured the occurrence and number of fall/s in the previous 12 months, the occurrence of fall-related injury/ies, and fall-related hospitalisations and/or medical treatment. Preventative behaviours (e.g., physical activity, health service consultations, safe footwear, calcium intake, and home modifications), attitudes and knowledge related to falls and physical activity, demographic and health-related factors also were measured; campaign awareness was assessed in the post-intervention survey only. Survey items were repeated verbatim in the baseline and post-intervention surveys in most cases.

Approximately 1600 persons were surveyed both at baseline and post-intervention in each of the two intervention sites. This sample size was deemed adequate to permit detection of relative differences between the pre- and post-intervention surveys of 15% in self-reported incidence of falls (ie, from 30% to 25.5%) and relative differences of 50% in incidence of hospitalisations due to fall injuries (from 3% to 2%), assuming 80% power and type I error of 5% (two-tailed). Incidence estimates were based on the original SOYF results [12]. In most cases, power was even better for the impact evaluation related to awareness, risk factors, and behaviours.

Due to the cross-sectional nature of the two surveys, conventional logistic regression models were used to consider the effects of interventions on each of the main outcomes of self-reported falls and injuries. Models were adjusted for potential confounding variables, including sex, age-group, education and general health as well as potential interactions between time and region, gender or age (to allow for differing intervention effects over time in the two locations, for males and females, or by 5-year age-group). Similar analyses incorporated separate logistic models for each attitude and behaviour related to falls prevention. All outcomes in these analyses are dichotomous variables.

### Ethics

The research was carried out in compliance with the Helsinki Declaration. Ethics approval for all aspects of the study was obtained prior to study commencement from the University Human Research Ethics Committee at Queensland University of Technology. Informed verbal consent was obtained from all survey participants prior to commencing their telephone interviews.

## Results

### Mortality and Morbidity

**Figure 1:**
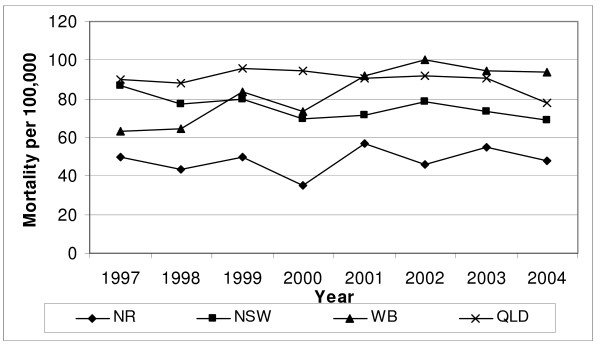
Mortality rates for fall or related fracture as underlying or contributory cause of death for adults aged 60 years or older in Wide Bay (Wide Bay) and Northern Rivers (Northern Rivers) regions in comparison to statewide rates for Queensland (Qld) and New South Wales (NSW) **. ** Used the average of the Australia population between 1997 and 2004 for standardisation of rates (60-, 65-, 70-, 75-, 80-, 85+ years)

Figure 1 displays the annual, age-standardised mortality rates due to falls for those aged 60 years or older from 1997 to 2004 for Wide Bay and QLD as well as Northern Rivers and NSW. There was considerable annual variation due to relatively small numbers, particularly for Wide Bay. Nevertheless, overall there was little difference when a fall was coded either as an underlying or contributing cause of death (data not shown). Although the average annual age-standardised mortality rates throughout the period were 23.2 and 23.3 per 100,000, respectively, for Wide Bay and QLD, this obscures a more recent increase in fall-related mortality since the beginning of the intervention in Wide Bay. During the last three years for which data are available, Wide Bay's mortality rate due to falls was approximately 10% higher than the QLD state rate for the same time. In contrast, the average annual age-standardised mortality rates were 19.5 per 100,000 for NSW and 13.4 per 100,000 for Northern Rivers. Northern River s' rate remained lower than the state-wide rates throughout the time period, and was on average 50% lower during the last three years for which data were available.

**Figure 2:**
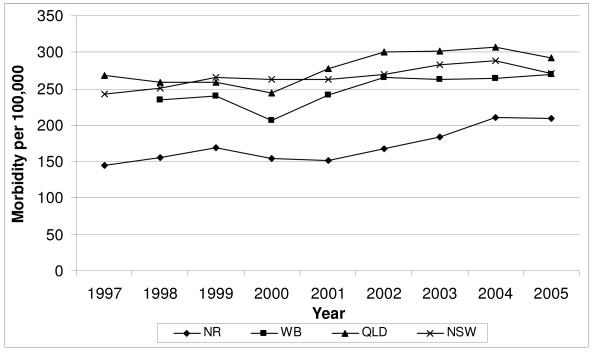
Hospitalisation rates for fall or related fracture for adults aged 60 years or older in Wide Bay (Wide Bay) and Northern Rivers (Northern Rivers) regions in comparison to statewide rates for Queensland (Qld) and New South Wales (NSW) **. ** Used the average of the Australia population between 1997 and 2004 for standardisation of rates (60-, 65-, 70-, 75-, 80+ years)

The average annual age-standardised morbidity rates for fracture with a fall coded as an external cause also were quite similar for the two states, at 146.0 per 100,000 for NSW and 148.3 per 100,000 for QLD from 1997-2005. However, again, Northern Rivers experienced a lower rate of hospitalisation throughout the time period at 91.3 per 100,000 compared to 128.8 per 100,000 for Wide Bay, although both locations showed increases in fall-related mortality starting around 2001. Most noteworthy, the morbidity rate in Northern Rivers was roughly 70% lower than its state-based rates before 2000, but was only 33% lower during the last couple of years of the intervention period (Figure 2). Trends were similar when analysed separately for men and for women, although women had much higher rates of fall-related fractures (data not shown).

### Survey responses, self-reported falls and risk factor prevalences

Response rates were between 73% and 81% at baseline and between 75% and 87% following intervention in Wide Bay, and ranged from 67% to 78% at baseline and 82% to 90% post-intervention in Northern Rivers, depending on whether households that refused prior to determining eligibility were included or excluded from the denominator. Since many of the refusing households likely did not have an age-eligible resident, response rates may be toward the higher end of estimates.

Differences in demographic and health characteristics between the pre- and post-intervention survey samples in both Wide Bay and Northern Rivers were not statistically significant for gender, general health status (Table 1), pension or private health insurance status (data not shown). In both regions, there were modest but statistically significant differences for level of education, partner status and employment status (Table 1). Post-intervention respondents were less likely to have a partner, slightly more likely to be retired, and less likely to report home-duties/caring as their current employment. Categories for education were inadvertently different in the pre- and post-intervention surveys hence individuals with a trade but no certificate may have nominated trade/technical at pre-intervention but junior high school at post-intervention.

**Table 1:** Characteristics of participants in the pre- and post- intervention surveys (2002 and 2006, respectively) from Wide Bay, Qld, and Northern Rivers, NSW

	Wide Bay					Northern Rivers				
	Pre		Post			Pre		Post		
Demographic	N	%	N	%	p-value	N	%	N	%	p-value
Gender					0.78					0.86
Male	801	50.0	818	50.5		800	50.0	811	49.7	
Female	800	50.0	801	49.5		801	50.0	822	50.3	
Age-group					0.011					0.001
60-64 yrs	396	24.8	454	28.2		335	21.0	408	25.1	
65-69 yrs	394	24.6	399	24.8		369	23.1	341	21.0	
70-74 yrs	351	22.0	295	18.3		339	21.2	297	18.3	
75-79 yrs	249	15.6	216	13.4		301	18.9	268	16.5	
80-84 yrs	127	7.9	158	9.8		150	9.4	204	12.6	
85+ yrs	82	5.1	89	5.5		102	6.4	107	6.6	
Education					<0.001					<0.001
University	126	7.9	148	9.2		189	11.8	191	11.8	
Trade/technical	408	25.6	245	15.2		433	27.1	270	16.7	
Senior high-school	129	8.1	146	9.1		133	8.3	160	9.9	
Junior high-school	317	19.9	424	26.4		396	24.8	522	32.3	
Primary or less	613	38.5	644	40.1		444	27.8	474	29.3	
Employment status					<0.001					<0.001
Retired	1186	74.2	1285	79.4		1169	73.0	1297	79.6	
Employed/student/volunteer	202	12.6	215	13.3		212	13.2	234	14.4	
Home duties/carer	158	9.9	73	4.5		180	11.2	61	3.7	
Unemployed/not working	52	3.3	46	2.8		40	2.5	38	2.3	
General health					0.93					0.31
Excellent	228	14.3	235	14.5		245	15.4	228	14.0	
Very good	454	28.5	470	29.1		486	30.5	525	32.2	
Good	506	31.7	517	32.0		528	33.1	515	31.6	
Fair	304	19.1	288	17.8		269	16.9	273	16.8	
Poor	102	6.4	106	6.6		67	4.2	88	5.4	

During both surveys, individuals were queried about a number of fall-related outcomes during the prior 12 months. The proportions of individuals reporting any fall, multiple falls, injury from a fall, hospital admission or medical attention because of a fall were fairly similar at pre- and post-intervention, with none of the outcomes showing statistically significant differences in separate models comparing pre- and post-intervention responses (Table 2). However, there was a statistically significant interaction for gender (p < 0.05), with fewer women reporting multiple falls during the prior 12 months between 2002 and 2006 (OR = 0.71; 95% CI: 0.54-0.95) and men showing virtually no change in reports of multiple falls during the same time (OR = 1.08; 95% CI: 0.80-1.48).

**Table 2:** Change in proportions of respondents reporting fall outcomes from pre- to post-intervention

**Outcome factor**^1^	%pre	%post	OR	95% CI
Fall in last 12 months	24.4	24.6	1.03	(0.92,1.16)
Multiple falls^2,3^	45.2	43.2		
Men	44.9	48.8	1.08	(0.80,1.48)
Women	45.5	38.4	0.71	(0.54,0.95)*
Injury from fall	16.6	15.7	0.95	(0.83,1.09)
Hospital admission	1.9	1.9	1.02	(0.71,1.47)
Medical attention	9.2	8.5	0.93	(0.78,1.11)

*p ≤ 0.05, **p ≤ 0.01, ***p ≤ 0.001

^1^Adusted for gender, age-group (60-64, 65-69, 70-74, 75-79, 80-84, 85+), education (university/college, trade/technical, senior, junior, primary or less), self-perceived general health (excellent, very good, good, fair, poor), region (Northern Rivers, Wide Bay)

^2 ^Results based on those reporting at least one fall in the last 12 months only

^3 ^Significant time × gender effect

Similar analyses were conducted for each of the 12 fall-related risk factors, many of which showed statistically significant improvements over time (Table 3). Three factors dealt with respondents' reports of discussions with health professionals. Discussions related to falls and medication side-effects were reported more frequently in 2006 than in 2002 (OR = 1.16; 95% CI: 1.03-1.31 and OR = 1.31; 95% CI: 1.14-1.49). However, the proportion of respondents reporting discussions with health professionals about physical activity did not increase, despite it being the primary focus of the interventions. Similarly, there was little evidence of increased awareness of media attention to falls or physical activity between 2002 and 2006, with a small decrease in awareness of falls messages and a similar increase in awareness of council activities related to falls among men but not women (interaction p < 0.05).

**Table 3:** Change in proportions of respondents reporting fall-prevention behaviour from pre- to post-intervention

Risk factor	%pre	%post	**OR**^1^	95% CI
Discussed falls with health professional	16.1	19.6	1.31	(1.14,1.49)***
Discussed activity with health professional	44.8	45.7	1.08	(0.98,1.20)
Discussed medications with practitioner	65.2	68.1	1.16	(1.03,1.31)*
Media awareness: falls	40.1	37.0	0.89	(0.81,0.99)*
Media awareness: physical activity	63.0	63.1	1.04	(0.94,1.16)
Awareness of falls-related council activity^2^	25.1	26.6		
Men	27.6	31.4	1.22	(1.04,1.43)**
Women	22.7	21.8	0.97	(0.82,1.15)
Safe shoes daily or almost daily	77.1	81.6	1.31	(1.15,1.48)**
Home modifications	28.1	32.9	1.29	(1.15,1.45)***
3+ serves of calcium	16.5	24.6	1.67	(1.47,1.89)***
Meeting moderate/vigorous guidelines	61.4	63.9	1.14	(1.02,1.27)*
Strength activity (2 days a week or more)	16.0	18.6	1.24	(1.08,1.41)**
Belief that falls are preventable^3^	61.4	63.9		
Northern Rivers	64.9	64.5	1.32	(0.76,2.30)
Wide Bay	57.9	63.2	0.99	(0.54,1.83)
Self-perceived low falls risk	62.4	60.9	0.94	(0.84,1.05)

Belief that falls are preventable^4^	61.4	63.9		
85+ years	47.8	49.5	1.03	(0.55,1.93)
80-84 years	42.4	56.8	2.17	(1.31,3.57)**
75-79 years	57.4	59.5	1.27	(0.88,1.85)
70-74 years	61.6	65.2	1.44	(1.04,2.00)*
65-69 years	64.9	67.6	1.21	(0.90,1.63)
59-64 years	70.8	68.3	1.02	(0.75,1.38)

*p ≤ 0.05, **p ≤ 0.01, ***p ≤ 0.001

^1^Adusted for gender, age-group (60-64, 65-69, 70-74, 75-79, 80-84, 85+), education (university/college, trade/technical, senior, junior, primary or less), self-perceived general health (excellent, very good, good, fair, poor), region (Northern Rivers, Wide Bay)

^2 ^Significant time × gender effect

^3^Significant time × region effect

^4 ^Significant time × age-group effect

Three factors commonly associated with reduced risk of falls or fall-related injuries were assessed, and all three were reported more frequently in 2006 than in 2002. Respondents were 31% more likely to report wearing safe shoes daily or almost daily, 29% more likely to report making home modifications to improve safety, and 67% more likely to report consuming 3 or more servings of calcium per day (p < 0.01 for each). There was an interaction between time and gender for calcium consumption that was of borderline statistical significance (interaction p = 0.068), reflecting an increase among women that was almost double that among men (OR = 1.41; 95% CI: 1.17-1.72 for men and OR = 1.85; 95% CI: 1.57-2.19 for women). With respect to physical activity, there was evidence of a 14% increase in the number of survey respondents reporting activity levels that met or exceeded national guidelines (30 minutes per day of moderate- or vigorous-intensity activity on most days of the week) (p < 0.05) and a 24% increase in those reporting strength-based exercise on two or more days per week (p < 0.01).

The majority of men and women in both locations and in all age groups continued to believe that their personal risk of falling was low, similar to levels in 2002. Perhaps of greater importance with respect to long-term prospects, those aged between 65 and 84 years showed 20% or greater increases in the number of respondents who reported believing that falls are preventable, with a doubling of individuals 80-84 years reflecting this view (OR = 2.17; 95% CI: 1.31-3.57). Those in the youngest (59-64 years) and oldest (85+ years) age groups showed little change over time with regard to this belief (interaction p < 0.01).

There was little difference between the two communities in terms of changes in falls risk factors. There was some improvement in the recognition that falls are preventable among Wide Bay residents, which although not statistically significant, at least brought levels closer to those reported among Northern Rivers residents, which remained fairly consistent across the intervention period (interaction p < 0.02). Wide Bay also achieved significant improvement in the prevalence of people discussing falls prevention with health professional. No significant difference was observed between the two communities in terms of changes in physical activity pre and post intervention.

## Discussion

The interventions in neither community substantially decreased the rate of falls-related injury among people aged 60 years or older, although there was some evidence of reductions in occurrence of multiple falls reported by women. In addition, there was some indication of improvements in fall-related risk factors, but the magnitudes were generally quite modest and some of these most likely reflected secular trends as they were not specifically targeted in the interventions. Most noteworthy is the considerable room for improvement still evident, even for the primary factors that were the focus of this intervention.

We chose to prioritise the strategy of increasing physical activity on the basis of the recent literature arguing that increased physical activity was an effective, scalable, population based intervention [13]. However there is evidence that increased physical activity increases the exposure of older persons to falling and while decreasing the falls risk per activity time, might increase the overall rate of falling in the population [26]. This may explain the lack of apparent effect of the intervention in our study.

The main difference between the current research program of SASI-SOYF and the original SOYF program relates to the intensity and comprehensiveness of the intervention. This difference enabled the study to evaluate whether a less ambitious, more streamlined (and potentially more sustainable and cost-effective), population-based, multi-factorial intervention could deliver comparable outcomes to the original, more intensively resourced study. Two models of streamlined intervention were examined in this study, a bottom up community development model and a top down structural change model. The lack of significant effect of either of the interventions in this study suggests that intensity of intervention is an important pre-requisite for success. This interpretation is supported by a recent meta-analysis of 19 studies similarly suggests that beneficial effects may be stronger for more intensive interventions [27].

Injury prevention projects delivered to whole populations are complex social policy interventions, which by definition are upstream activities that aim to influence the situations in which the causal components of injury may potentially occur [28]. However, falls prevention activities are generally conceived as small projects with limited timelines. They operate within one or two domains of the *Ottawa Charter for Health Promotion *[29] with minimal tangible commitment or collaborative planning among key stakeholders and no dedication to recurrent resourcing. Population-based interventions generally adopt bottom-up, community development approaches. There is growing evidence that bottom-up interventions are not in themselves effective solutions to the problem of injury [30,31]. While local involvement is critical to the success of population-based interventions, effective action to prevent injury does require orchestrated support from the comparatively well-resourced and empowered societal leadership. This maximises and amplifies the outcomes of local initiatives with changes to the social institutions in which causal events, conditions and attributes are created and sustained.

The evidence provided by the evaluation of *'Stay Active, Stay Independent, Stay on Your Feet' *calls for the coordinated implementation of high intensity public health programs. Population health outcomes cannot be achieved by implementing multiple projects of limited scope focusing on different issues for short periods. Major successes in the past, e.g., tobacco control and use of seat belts, have required extensive and prolonged attention with interventions ultimately engaging all aspects of society, including alteration of legal and cultural norms. An analogous effort relates to influencing water consumption during the recent, and for much of Australia, ongoing drought. Improvement in public health will not be achieved if funders and policy-makers are distracted by one seemingly enormous public health issue after another. In the context where population health interventions cannot hope to cover all areas of need, the burden-of-disease approach provides the rationale for restricting activity to those areas for which improvement will make the biggest difference to the health of the population. This approach is an effective solution for the leading causes of population morbidity and mortality, because the causal pathways for most health outcomes including falls, injury, cancer, and many chronic health conditions, are remarkably similar.

## Conclusions

The implementation of a generic prevention approach will reduce the intrinsic risk factors across the whole population before they manifest themselves as proximal risk factors for falls. Up-stream solutions that enable individual and societal behaviours that entrench physical activity, healthy nutrition, the elimination of smoking, moderate alcohol consumption, and active social involvement from an early age are as much evidence-based prevention strategies for falls injury as they are chronic disease prevention strategies [32]. If government and community sectors focus population resources on the reduction of generic distal risk factors, and clinical resources on proximal risk factors, the continuum of risk will be more effectively addressed and finite resources more efficiently deployed. It is argued that if such an orchestrated intervention were effectively implemented over a sustained period, a reduction in the population-level indicators of falls injury among older persons would follow.

## Competing interests

The authors declare that they have no competing interests.

## Authors' contributions

All the authors made an important contribution to the development of the ideas and conduct of the research. RM, KH and BN were primarily responsible for developing the first drafts of the manuscript; KH, KM and CR made a major contribution to the analysis; ED, UD and PV contributed to refining the concepts and content comprising the final manuscript. All authors have been integrally involved in reviewing and extensively editing the text as it has progressed through several iterations.

## Pre-publication history

The pre-publication history for this paper can be accessed here:

http://www.biomedcentral.com/1471-2458/10/79/prepub
